# Redefining and Identifying Evidence-Based Indications for Open Reduction and Internal Fixation in Mandibular Condylar Fractures: A Comprehensive Systematic Review and Evidence Analysis

**DOI:** 10.3390/cmtr18020025

**Published:** 2025-04-22

**Authors:** Stephen A. L. Y. Youssef, Iva I. Raghoebar, Renee Helmers, Jan de Lange, Leander Dubois

**Affiliations:** Department of Oral and Maxillofacial Surgery, Amsterdam University Medical Center, University of Amsterdam, Meibergdreef 9, 1105 AZ Amsterdam, The Netherlands; i.i.raghoebar@amsterdamumc.nl (I.I.R.); r.helmers@amsterdamumc.nl (R.H.); j.delange@amsterdamumc.nl (J.d.L.); l.dubois@amsterdamumc.nl (L.D.)

**Keywords:** mandibular condyle fracture, surgical treatment, open treatment, open reduction and internal fixation, indication, evidence analysis

## Abstract

A major controversy in maxillofacial surgery practice is the management of mandibular condylar fractures (CFs). The debate revolves around open versus closed treatment, rather than identifying clear indications whereby open reduction and internal fixation (ORIF) is the most viable treatment modality. Opinions regarding precise indications for ORIF remain unclear and non-uniform. We aimed to refocus the debate regarding the optimal treatment for CFs by identifying the recent indications for ORIF in the literature and assessing the quality of the existing evidence for each indication. This systematic review searched Medline, Embase and the Cochrane Central Register of Controlled Trials for eligible studies. The included studies consisted of articles from the past 15 years involving patients with any type of CF who underwent ORIF based on specified indications. From 4711 papers, 100 studies were included. In these, 121 indications were identified. The most cited indications for ORIF were those proposed by Zide and Kent, namely displacement/angulation ≥10° and ramus height shortening of ≥2 mm. Evidence supporting these indications is weak, relying mainly on expert opinion rather than robust data, with a focus on treatment comparisons. Clear, evidence-based cutoffs regarding when ORIF is the only viable treatment option are needed for a consensus.

## 1. Introduction

Condylar fractures are a common outcome of facial trauma and can present with clinical signs such as malocclusion, pain, limited jaw mobility and facial asymmetry. Long-term complications like temporomandibular joint (TMJ) disorders, ankylosis, malunion and persistent facial asymmetry can have a significant impact on a patient’s quality of life [1,2,3,4].

Given the significant impact of complications associated with condylar fractures on patients’ well-being, it becomes imperative to explore established guidelines and criteria for their management. Zide and Kent’s [5] research was pioneering, being the first to address this topic in the early 1980s. Their research established a comprehensive range of indications, including absolute criteria such as fractures displaced into the middle cranial fossa and lateral extracapsular displacement. Examples of the provided relative indications were bilateral condyle fractures in edentulous patients and cases where intermaxillary fixation and physical therapy were impractical. While these guidelines were beneficial in the early 1980s, they were developed based on the materials and surgical procedures that were available at that time [5,6].

Over time, several new insights were proposed in literature and built on these indicators. In their systematic review, Minervini et al. [7] summarized these indicators over time to determine when surgical or non-surgical treatment was appropriate. Despite the high incidence of mandibular condylar fractures in their study, a consensus regarding the most effective therapeutic approach remains elusive, contributing to significant clinical divergence. This is primarily due to the absence of standardized definitions of condylar fractures, leading to considerable heterogeneity among studies and the limited quality of the research. Consequently, many noncomparative studies have emerged, describing diverse approaches to addressing condylar fractures [7,8].

In the past, the ongoing debate has predominantly centered on comparing treatment modalities, such as CR versus ORIF, based on long-term outcomes and complications. However, significant differences within these studies have led to considerable controversy, which makes it difficult to reach a consensus [9,10]. In our view, further analysis of this comparison is of limited value. Rather than trying to determine which treatment is best for specific cases, we should refocus on identifying the situations or indications in which ORIF is the only viable therapeutic option. This alternative approach and shift in focus should contribute to clearer and evidence-based criteria for ORIF. The primary objective of this study is to systematically identify and review the indications in which ORIF is the most viable therapeutic option. We aim to achieve this by assessing the existing evidence and its quality, as well as the extent to which these indications are supported by clinical assessments, radiographic findings, or a combination of both.

## 2. Materials and Methods

### 2.1. Registration and Search Strategy

This systematic review was registered with PROSPERO (registration number: 1021504) and was conducted following established guidelines for systematic reviews. Systematic searches of the literature were performed in PubMed, Embase and Cochrane on 27 October 2023. The search strings (Table A1) for the previously mentioned databases were created with the help of a biomedical information specialist. After conducting the automated search and removing duplicates, a manual search process was undertaken to assess the eligibility of the publications. This systematic review fulfilled the Preferred Reporting Items for Systematic Reviews and Meta-Analyses (PRISMA) guidelines [11].

### 2.2. Eligibility Criteria

The eligible studies for inclusion consisted of articles published between 2009 and 2023, involving adult patients diagnosed with any type of condylar fracture who underwent ORIF based on the provided indications. Any justification provided to support the use of ORIF was considered an indication. Studies with patients with a similar fracture, where, despite initial indications for closed reduction (CR), a decision was made to perform ORIF, were also included.

The following study designs were considered eligible for inclusion in this review: systematic reviews (SR); literature reviews; randomized controlled trials (RCTs); and cross-sectional, observational, prospective cohort or retrospective cohort studies that met the required criteria and with a minimum of ten included patients. Additionally, ORIF indications from the current guidelines provided by specialty departments, accessible online, were also included in the review.

The following study exclusion criteria were used: case studies, expert opinions, reviews in which the analyzed studies had already been identified and included, animal studies, in vitro studies, pathological fractures, gunshot fractures, no reported indications for ORIF, and condylar fractures that were indistinguishable from other mandibular fractures.

### 2.3. Screening Methods

The titles and abstracts of the identified articles were screened by two independent reviewers (S.Y. and I.R.) for inclusion eligibility. All articles meeting the inclusion criteria underwent a comprehensive assessment through the acquisition of their full-text documents. In instances of doubt, each article underwent an independent full-text evaluation by both reviewers. The resolution of any discrepancies between the reviewers took place through discussion. If persistent disagreements arose concerning inclusion, a third reviewer, R.H., was available for consultation. The interrater reliability for title and abstract screening, along with full-text evaluation, was quantified using Cohen’s kappa coefficient (κ) and the percentage of agreement among the reviewers. Non-English articles were translated by a medically qualified native speaker proficient in both the language of the related article and English. If the full text was unavailable, the university’s information analyst and the study researchers were consulted.

### 2.4. Data Extraction

Articles identified through the search were screened to determine whether they cited any indications for ORIF. If so, the following data were extracted and compiled in a pre-defined form: author; publication year; study design; sample size and patient characteristics; follow-up period; number of patients treated with ORIF/CR; inclusion criteria for patients; the assessed outcomes of the studies and their significance; whether the results favored ORIF/CR; the definition of CR; whether the study’s objective was to validate an indication; the section in which the indication was mentioned (introduction; methods; results; discussion); the indication for ORIF that was used/mentioned; whether a management protocol was proposed; the proposed indications based on the findings; whether the CF was specified in any form (site of CF/type of CF and the classification method used); how displacement, angulation, or ramus height shortening was measured; how occlusion disorders were objectified; the limitations of the studies; the level of evidence according to the Oxford Clinical Medicine Levels of Evidence; and the outcome of the AMSTER2 tool assessment. Only indications cited five or more times and clearly specified were included in the comprehensive overview table (Table 1). A cutoff of five was used to provide a clear overview of the most frequently cited indications, emphasizing those with significant relevance and prominence in the literature.

### 2.5. Evaluation of Evidence, Risk of Bias and Study Quality

SRs, RCTs, prospective and retrospective studies that aimed to validate an indication, rather than merely mention it, were assessed for further critical analysis. The level of evidence analysis was executed using the Oxford Centre for Evidence tool [12] and referred to Table 1, based on the most frequent evidence level among the included studies. The risk of bias assessment was conducted using the Rob 2.0 and Robvis-I tools [13,14,15], specifically applied to the RCTs and prospective studies, as they are suitable for these study types, providing tailored, standardized bias evaluations. For the SRs, the quality was evaluated using the AMSTAR-2 tool [16].

## 3. Results

**Figure 1:**
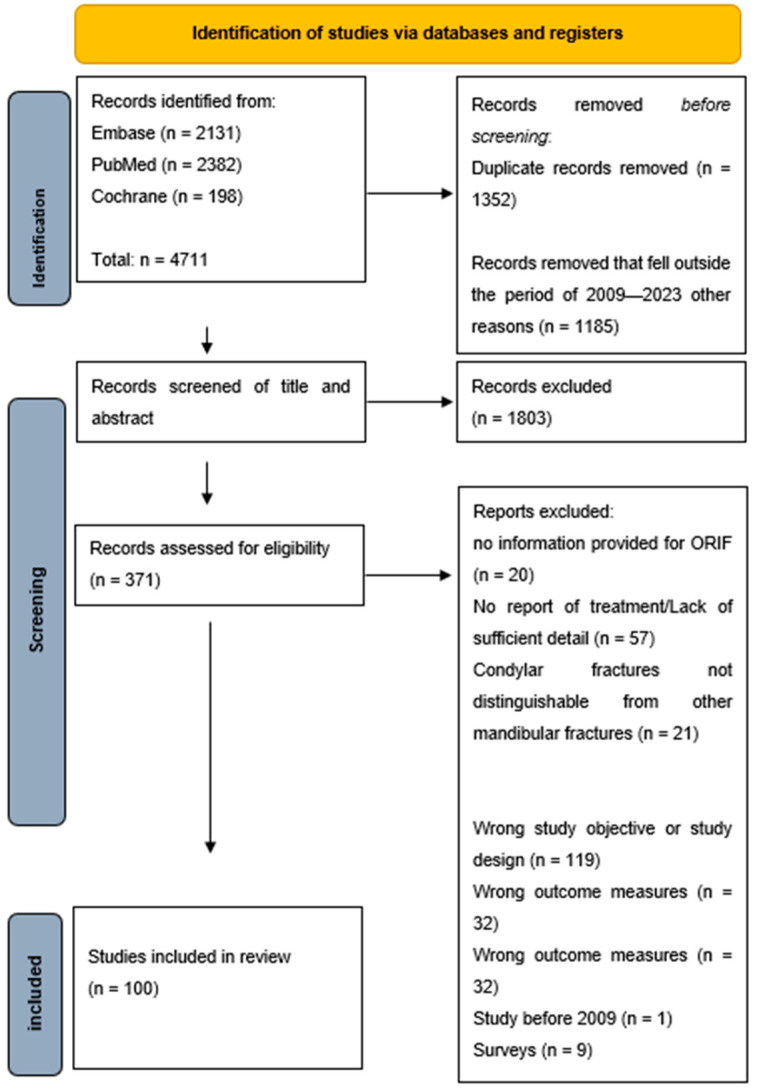
Flowchart of the study selection procedure.

The primary search, conducted on 27 October 2023, generated 198 hits in Cochrane, 2131 hits in Embase and 2382 hits in PubMed. Following the elimination of duplicates and records outside the predetermined timeframe of 2009–2023, a total of 2174 records remained eligible for title and abstract screening. Cohen’s kappa coefficient (κ) was 0.73, with a percentage of agreement of 86%. Subsequently, 371 manuscripts underwent comprehensive full-text analysis, leading to the inclusion of 100 manuscripts in the final review (Figure 1). Cohen’s kappa coefficient (κ) was 0.76, with a percentage of agreement of 88%. Eight manuscripts were translated by a native speaker.

A comprehensive overview of the indications identified in the literature is provided in Table 1, highlighting those cited in five or more articles from our search of publications over the past 15 years. In total, 121 indications were identified. Twenty indications were excluded from the list because they were cited in fewer than five instances in the search. Additionally, indications lacking sufficient specification were omitted due to their limited clinical relevance (Table 2).

Notably, Zide and Kent’s proposed indications were the most frequently mentioned across the articles, with a total of 42 citations, 30 of which lacked any attempt at validation. Among the validation studies, six were retrospective cohort studies, and none were prospective randomized studies. The evidence analysis yielded a result of level four according to the Oxford Clinical Levels of Medicine tool. Indications concerning displacement/angulation specified in degrees were cited 22 times. Among these, ORIF was recommended in cases of angulation of 10° or more in 15 instances. Of these 15, three citations lacked an attempt at validation. Among the validation studies, there were two retrospective cohort studies and five randomized prospective studies. The evidence analysis yielded a result of level 1b. Additionally, ramus height shortening was noted as an indication on 21 occasions, with ORIF deemed appropriate in 19 cases where the shortening exceeded 2 mm or more. In the other two cases, shortening of 15 and 17 mm or more was deemed appropriate for ORIF. Of these 19 cases, eight citations lacked an attempt at validation. Among the validation studies, there were two retrospective cohort studies and four randomized prospective studies. The evidence analysis yielded a result of level 1b.

A comprehensive overview of the articles aimed at validating an indication can be found in Table A2. In total, 27 articles aimed to validate one or more indications. These studies proposed 50 different indications for validation. All studies are presented in Table A2. The distribution of the evidence levels is as follows: 17% of studies are categorized as level 1b, 33% as level 2b, 29% as level 3b and 33% as level 4. The characteristics and indications of the prospective studies included in this systematic review are depicted in Table A3. Table A4 offers insights into the methodologies employed in the included SRs, the consulted databases, and comprehensive details regarding the included studies. Additionally, it includes the definitions of the included CFs and highlights any insufficient descriptions of these definitions.

The reporting of treatment outcomes across studies was inconsistent and scattered, leading to difficulties in data comparison. However, the most frequently reported outcomes included occlusion, mandibular deviation, the range of motion (ROM), maximum mouth opening (MMO), nerve dysfunction, ramal height restoration, temporomandibular joint (TMJ) function, chronic pain scores, functional questionnaire results, and esthetic outcomes.

In the analysis of the included thirteen prospective studies, ten studies (83%) reported the significant superiority of ORIF compared to CR. This assessment was based on the number of outcome measures that showed significant positive results for each method, with a greater proportion of measures favoring ORIF. None of these studies indicated a significant advantage for CR, although two studies (17%) reported no significant difference between the two methods. One study [17] could not be included as it did not present its results comparably (Table A5). Nine studies (69%) clearly described their study groups and the type of CF; only four studies (31%) performed a heterogeneity analysis, of which three studies did not show significant differences between the groups.

**Figure 2:**
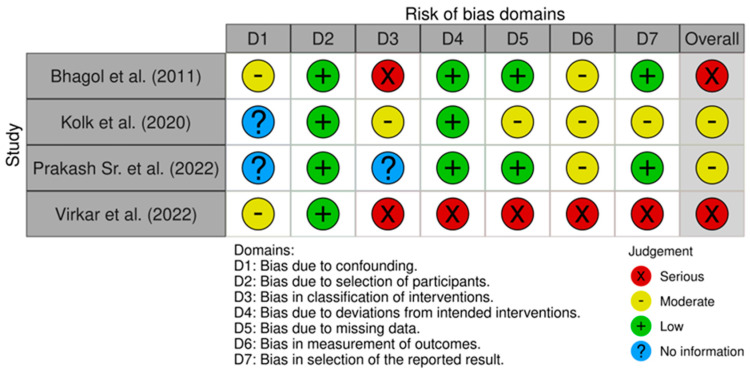
Risk of bias assessment—Robins-I tool—Bhagol et al. (2011) [18], Kolk et al. (2020) [19], Prakash Sr. et al. (2022) [20], Virkar et al. (2022) [21].

**Figure 3:**
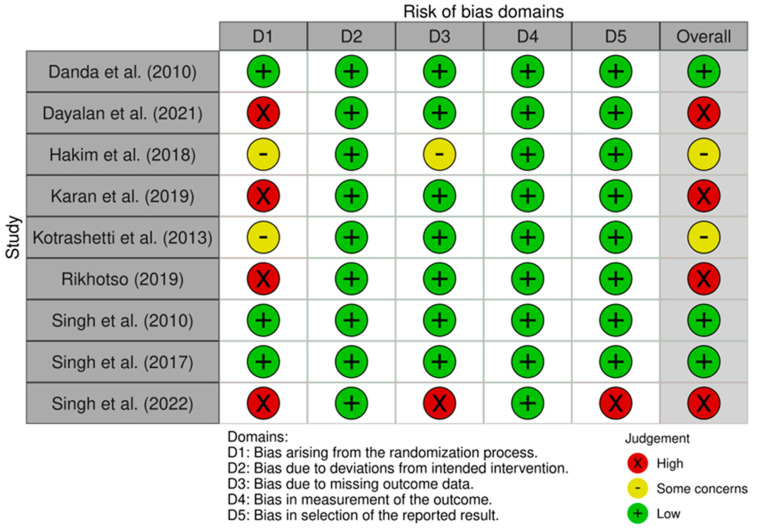
Risk of bias assessment—RoB 2.0 tool—Danda et al. (2010) [22], Dayalan et al. (2021) [23], Hakim et al. (2018) [24], Karan et al. (2019) [25], Kotrashetti et al. (2013) [26], Rikhotso et al. (2017) [27], Singh et al. (2010) [28], Singh et al. (2018) [29], Singh et al. (2022) [17].

Within the SRs, nine studies (78%) revealed the significant superiority of ORIF over CR. None of the studies reported the significant superiority of CR, while two studies (22%) found no significant difference. Two studies were excluded from consideration: one due to solely presenting ORIF results and the other due to providing only a treatment protocol, without showing any results (Table A6). Three SRs (33%) included homogeneous study designs in their reviews, and only two reviews (17%) specified a specific site or type of CF. Figure 2 and Figure 3 show the risk of bias assessments of these studies.

Table A7 provides a comprehensive overview of the outcome interpretations by the authors in prospective studies, the proposed indications based on their findings, and the treatment algorithm specifications, limitations, and levels of evidence. The majority of the authors advocate for ORIF based on their findings, with one study [30] specifying a distinct treatment approach. The distribution of the evidence levels is as follows: 38% of studies are categorized as level 1b, 54% as level 2b and 8% as level 3b. Table A8 provides a similar comprehensive overview to that of the SRs, with the addition of the AMSTAR-2 assessment. Overall, 89% of the studies advocate for ORIF and 0% for CR; 11% were not able to make a conclusive statement on this matter. Two studies specified a treatment approach. The distribution of the evidence levels is as follows: 25% of studies are categorized as level 1a, 67% as level 2a and 8% as level 3a. According to the AMSTAR-2 tool, the quality of the reviews was high in 25%, moderate in 33%, low in 8% and critically low in 33%.

## 4. Discussion

Rather than focusing on the outcomes of open versus closed therapy, this review aimed to redirect attention toward the true indications for ORIF, aiming to establish clear cutoff points where ORIF is the most effective treatment option. This aim could not be fully achieved due to persistent inconsistencies in the literature and due to the approach within the literature, whereby identifying cutoff points for when ORIF is the most effective treatment are rarely considered. A comprehensive summary of the latest evidence-based advancements and an assessment of the quality of these studies were provided through a critical analysis of the scientific evidence collected over the past 15 years. The original studies that form the basis of the currently identified indications were reviewed to assess the quality of the evidence.

Regardless of the extensive research on the topic, the consensus regarding indications for ORIF is primarily based on expert opinion and common sense, rather than high-quality evidence. The generally accepted indications in the literature, as stated by Zide and Kent [5], were mainly derived from case reports and literature reviews. It is remarkable that these have been the most frequently cited indications for ORIF in CFs in the past 15 years. The consensus seems to be that severely displaced fractures should be treated with ORIF [25,31,32,33]. However, a consensus is lacking regarding the criteria defining a displaced fracture, as the literature proposes varying cutoff points. These cutoff points are frequently derived from the surgeon’s clinical experience, and their validation has been attempted in the literature, often with studies of insufficient quality [25,29,30,34,35,36].

Establishing an indication based on high-quality evidence is crucial in enhancing its strength and the probability of achieving a therapeutic goal [37]. It forms the ethical basis for treatment decisions and ensures rational patient care. The challenge in formulating indications for ORIF based on high-quality evidence lies, among others, in the comprehensiveness and complexity of CF management. Assael et al. identified [29] widely accepted variables among clinicians that are all considered in the clinical decision-making for ORIF. Due to the lack of clear cutoff points, the weighting of these variables in making a clinical decision regarding the need for ORIF remains an empirical art [38].

The cited and investigated indications described in the past 15 years seem to mainly focus on two-dimensional radiological variables [18,26,28,30], aiming to establish cutoff points for ORIF. It is specifically the three-dimensional radiological analysis that offers more insights into the healing morphology and the adaptation mechanisms of the condyles [39,40]. Clinical variables receive less attention since these are frequently only mentioned for consideration and not described in detail. To illustrate this, occlusion disorders are frequently reported but are rarely defined, complicating the ability to draw conclusions from the reported results and to compare these with other study results.

Another complicating factor is the lack of consistency in describing CFs. Various classification systems have emerged, each describing CFs differently, with a lack of consensus on which classification system to use [41,42,43,44,45,46,47,48]. Neff’s attempt to establish an overarching classification was hindered by overly complex and detailed descriptions of condylar fractures, thereby serving more as an illustrative classification rather than a classification that serves as a guide in therapeutic decision-making [43]. The variability in these classifications describing CFs differently results in a high degree of heterogeneity among CFs. This makes it very challenging to compare research and to draw conclusions. Consequently, meta-analyses comparing mainly functional aspects are not able to specify the levels and sides of CFs, nor do they detail the degree of displacement, ramus height shortening or clinical parameters [10,49,50,51,52,53,54,55]. The same applies to the identified prospective studies, in which a quarter of the studies do not describe their CF groups, thereby making their results difficult to put into perspective. Therefore, it is impossible to clinically determine in which cases ORIF is suitable for a particular CF subtype and to determine where the cutoff point is for a specific indication.

The primary focus of the literature over the past 15 years regarding the indications for ORIF is summarized in Table 1. Angulation and ramal height shortening were by far the indications for which validation was most frequently attempted. It is remarkable that, in 80% of these studies, ramal height shortening of ≥2 mm was considered as an indication for ORIF [18,24,25,28,56,57]. However, no substantiation of this 2 mm cutoff could be found in the literature. Articles frequently refer to the studies by Eckelt, Schneider, and Singh et al. [28,58,59], where ORIF demonstrated superior results across all objective and functional parameters. However, none of these authors provide clear rationale for the 2 mm reduction in the ramus height, despite emphasizing the consensus in the literature on the importance of the correct anatomical reconstruction of the condylar process to restore function. Furthermore, their studies did not investigate whether the 2 mm cutoff point led to better outcomes than different cutoff points for ramus height shortening [28,58,59,60]. The RCT conducted by Rikhotso et al. [27] was the only study to illustrate the effect of increased ramus height loss on outcomes. Rikhotso et al. demonstrated that the odds of complications increased by 1.26 for every 1 mm increase in height. According to the findings, the likelihood of complications surpasses 50% when the vertical height loss exceeds approximately 5 mm. Based on the available evidence in the literature, ramus height shortening of more than 2 mm alone is insufficient as an indication for ORIF [27].

The body’s ability to adapt to new conditions is shown in condylotomies, where up to 8 mm of ramus shortening does not typically result in functional problems [61,62]. Helmer et al. [63] showed, in a finite element model study on load distribution following unilateral condylar fractures, that it is not the sudden ramus height loss that leads to complications, but rather the abrupt changes in load distribution. It was demonstrated that the load distribution was unfavorable beyond 6 mm of ramus height loss and likely to cause functional complications [63,64]. The consensus to aim for anatomical repositioning for optimal functional outcomes seems outdated in this context. The focus should shift to defining a cutoff point based on function rather than anatomical restauration. The efforts of Helmer et al. [63,64,65] and Rikhotso et al. [27,30] are pioneering in defining this cutoff point based on robust evidence with high quality.

Regarding angulation, Rikhotso et al. [27] indicated that the probability of complications increases as the angle of displacement exceeds 15°. Helmer et al. [65] suggested that a fracture angle greater than 18.75° leads to an increased load on the unaffected side and a decreased load on the affected side, possibly resulting in clinical TMJ dysfunction. In angulated CFs without ramal shortening, an increase in contact stress was seen at angulation of 15° and 35°, with the stress centralizing at larger angles. This indicates minimal remodeling at higher angulation, with 15° as a potential cutoff point [27,63,64,65].

The literature lacks standardized measurement methods for angulation and ramus height reduction. The identified articles often did not describe the measurement methods. This raises questions such as whether the displacement of the fracture gaps was accounted for and whether the contralateral side was used as a reference or the fractured side was measured. Additionally, what specific measurement points were utilized? In the study of Kommers et al. [66], it was demonstrated that two-dimensional imaging is susceptible to intra- and inter-observer errors. Additionally, it was noted that fractures sometimes result in an increase in ramus length compared to the unfractured contralateral side, further complicating the reliable measurement of reference heights. Developing standardized and validated measurement techniques is crucial to accurately define CF groups and establish homogeneous research cohorts for meaningful comparisons.

In conclusion, the evidence from the past 15 years supporting true indications for ORIF is substantially weak. Despite the considerable heterogeneity in the description of condylar fractures and the lack of consistent and validated measurement techniques, the literature continues to focus on finding the best possible therapy, particularly comparing CR versus ORIF. Primarily due to this factor, the progress towards a consensus seems of limited extent. Although no new indications have been identified, we believe that this review refocuses the discussion on these indications by assessing whether they can serve as cutoff points regarding when ORIF is the most viable therapeutic option. This refocus results in questioning the already implemented indications for ORIF based on the lack of clarity regarding their scientific foundation. In the past 15 years, the tendency in the academic literature has been influenced by the reliance on expert opinion, subsequently leading to cutoff values that lack robust empirical support for ORIF indications.

Due to the heterogeneity in classifying CFs, it is challenging to draw definitive conclusions from the research results in the literature. Standardizing verified measurement methods, combined with the development of a clinically applicable, standardized classification system, should contribute to greater homogeneity in the types of CFs, allowing the outcome measures to be placed into perspective.

The literature would greatly benefit from research focusing on the threshold where ORIF becomes preferable. Defining this cutoff based on factors like complexity, recovery outcomes and complications would guide evidence-based decisions and improve treatment strategies. Similar studies to those conducted by Helmer et al. and Rikhotso et al. are necessary to develop reliable cutoff points that can serve as indications for ORIF.

## Figures and Tables

**Table 1:** General overview of indications found in literature based on 5 or more citations in our literature search of all articles published in the last 15 years.

Indication Used for ORIF		Number of Times Cited	Number of Times Cited Without Validation or Based on Expert Opinion	Retrospective Studies with Control Groups	Prospective Randomized Studies	Overall Level of Evidence (Assessment According to Oxford)
Zide and Kent’s criteria		42 (35%)	30 (71%)	6 (85%)	0 (0%)	4
Degree of angulation	angulation ≥ 10 degrees	15 (12%)	6 (40%)	2 (100%)	5 (80%)	1b
	angulation ≥ 20 degrees	4 (3%)	3 (75%)	0	1 (100%)	2b
	angulation ≥ 45 degrees	3 (2%)	2 (67%)	0	0 (0%)	1a
Ramus height shortening	≥2 mm	19 (16%)	8 (42%)	2 (100%)	4 (100%)	1b
	≥15 mm	1 (1%)	1 (100%)	0	0	-
	≥17 mm	1 (1%)	1 (100%)	0	0	-
Necessity of stable mandible for other fractures/mid-facial fractures associated with CF/displacement in CF with malocclusion or mid-facial fracture		13 (11%)	11 (85%)	1 (50%)	0	-
Bilateral CF with or without other facial fractures		8 (7%)	4 (50%)	2 (100%)	1 (100%)	4
Inferior dislocation of condylar head + ramus shortening/displaced, intra-capsular fractures, with decrease in ramus height		5 (4%)	1 (20%)	3 (100%)	1 (33%)	3b
MMF not feasible		5 (4%)	4 (80%)	0	0	2b
AAOMS criteria		5 (4%)	4 (80%)	1 (100%)	0	4 (occlusion: 1a)

CF: condylar fracture; AAOMS: American Association of Oral and Maxillofacial Surgery; MMF: maxillomandibular fixation.

**Table 2:** Overview of excluded indications due to less than 5 citations.

Indication	Number of Times Cited
Unilateral CF without contact of fracture segments	1
Bilateral CF without contact of fracture segments	2
CF with proximal segment > or = 2cm	1
Bilateral CF with orthognathic position abnormalities	2
Type B (Neff’s classification) fractures	1
Type III Wits classification (Author’s own classification)	1
Anteromedial displacement of the discus/tearing of discus	2
Pain in the region during mandibular movements	1
MacLennan classification 3 or higher	1
Positive Ellis III drop back test	1
Involvement of capsular and disco-ligamentous soft tissues of the temporomandibular joint	1
Bony overlap of more than 5 mm	2
Open wound fractures	4

CF: condylar fractures.

## Data Availability

No new data analyses were produced in this study.

## Abbreviations

The following abbreviations are used in this manuscript:

CF	Condylar fractures
ORIF	Open reduction and internal fixation
TMJ	Temporomandibular joint
PRISMA	Preferred Reporting Items for Systematic Reviews and Meta-Analyses
CR	Closed reduction
SR	Systematic review
RCT	Randomized controlled trial
ROM	Range of motion
MMO	Maximal mouth opening

## Appendix A

**Table A1:** Search strategy.

Database	Strategy
Medline/PubMed	((“Surgical Procedures, Operative”[Mesh] OR “Fracture Fixation, Internal”[Mesh] OR Surgical procedure*[tiab] OR Surgical treatment*[tiab] OR Operative Procedure*[tiab] OR Operative Treatment*[tiab] OR Maxillofacial Procedure*[tiab] OR Maxillofacial Treatment*[tiab] OR ORIF[tiab] OR “Internal fixation”[tiab] OR Open reduction[tiab] OR Open treatment*[tiab] OR Internal Fracture Fixation*[tiab] OR Fracture Osteosynthes*[tiab] OR “conservative treatment”[MeSH Terms] OR “conservative*”[tiab] OR “nonoperative*”[tiab] OR “non operative*”[tiab] OR “nonsurgical*”[tiab] OR “non surgical*”[tiab] OR “non invasive”[tiab] OR “noninvasive”[tiab] OR “Closed therap*”[tiab] OR “Closed Treatment*”[tiab] OR “Closed reduction*”[tiab]) AND (“Mandibular Condyle”[Mesh] OR “Mandibular Fractures”[Mesh] OR Mandibular Condyle*[tiab] OR Mandibular Fracture*[tiab] OR “Condylar Fracture”[tiab:~2] OR “Condylar Fractures”[tiab:~2])) AND (Indication*[tiab] OR Criteri*[tiab] OR Management[tiab] OR Approach*[tiab])
Embase	(Surgical treatment* or Operative procedure* or Maxillofacial Procedure* or Maxillofacial Treatment* or ORIF or “Internal fixation” or Open reduction or Open treatment* or Internal Fracture Fixation* or Osteosynthes*).ti,ab,kf. or conservative treatment/or “conservative*”.ti,ab,kf. or “nonoperative*”.ti,ab,kf. or “non operative*”.ti,ab,kf. or “nonsurgical*”.ti,ab,kf. or “non surgical*”.ti,ab,kf. or “non invasive”.ti,ab,kf. or “noninvasive”.ti,ab,kf. or “Closed therap*”.ti,ab,kf. or “Closed Treatment*”.ti,ab,kf. or “Closed reduction*”.ti,ab,kf. or closed approach.ti,ab,kf. or closed management.ti,ab,kf. or closed technique*.ti,ab,kf. or closed method*.ti,ab,kf. AND Mandible/or Mandible Fracture/or Mandibular Condyle*.ti,ab,kf. or Mandibular Fracture*.ti,ab,kf. or Mandible Condyle.ti,ab,kf. or Mandible fracture*.ti,ab,kf. or (Condylar adj2 Fracture*).ti,ab,kf. or subcondylar fracture*.ti,ab,kf. AND (Indication* or Criteri* or Management or Approach*).ti,ab,kf. NOT Conference abstracts
Cochrane	([mh “Surgical Procedures, Operative”] OR [mh “Fracture Fixation, Internal”] OR (“Surgical” NEAR/2 procedure*):ti,ab,kw OR (“Surgical” NEAR/2 treatment*):ti,ab,kw OR (“Operative” NEAR/2 Procedure*):ti,ab,kw OR (“Operative” NEAR/2 Treatment*):ti,ab,kw OR (“Maxillofacial” NEAR/2 Procedure*):ti,ab,kw OR (“Maxillofacial” NEAR/2 Treatment*):ti,ab,kw OR “ORIF “Internal fixation”“:ti,ab,kw OR “Open reduction”:ti,ab,kw OR (“Open” NEAR/2 treatment*):ti,ab,kw OR (“Internal Fracture” NEAR/2 Fixation*):ti,ab,kw OR (“Fracture” NEAR/2 Osteosynthes*):ti,ab,kw OR [mh “conservative treatment”] OR conservative*:ti,ab,kw OR nonoperative*:ti,ab,kw OR (“non” NEAR/2 operative*):ti,ab,kw OR nonsurgical*:ti,ab,kw OR (“non” NEAR/2 surgical*):ti,ab,kw OR “non invasive”:ti,ab,kw OR noninvasive:ti,ab,kw OR (“Closed” NEAR/2 therap*):ti,ab,kw OR (“Closed” NEAR/2 Treatment*):ti,ab,kw OR (“Closed” NEAR/2 reduction*):ti,ab,kw) AND ([mh “Mandibular Condyle”] OR [mh “Mandibular Fractures”] OR (“Mandibular” NEAR/2 Condyle*):ti,ab,kw OR (“Mandibular” NEAR/2 Fracture*):ti,ab,kw OR Condylar NEAR/2 Fracture*) AND (Indication*:ti,ab,kw OR Criteri*:ti,ab,kw OR Management:ti,ab,kw OR Approach*:ti,ab,kw)

**Table A2:** Overview of all articles that aimed to validate an indication for ORIF in CFs.

Author and Year	Study Design	Inclusion Criteria	Sample Size	Examined Indication	Included CF Specified	Assessed Outcomes	Results Favoring ORIF/CR	Proposed Indication(s) for ORIF Based on Findings	Level of Evidence
B. Akinbami (2014) [67]	Retrospective study	Patients undergoing surgery of the condyle or ramus	2 (ORIF)	Gross displacement and extracapsular fracture	Not specified	MIO, nerve dysfunction, ROM	N/A	ORIF is favored in dislocated or displaced condylar fractures.	4
N. Dayalan (2021) [23]	Prospective study	Patients with isolated unilateral subcondylar fractures	20 (10 ORIF, 10 CR)	Isolated low unilateral subcondylar fracture with moderate displacement, with the condyle still seated in the fossa	Specified	Esthetic improvement, MIO, nerve dysfunction, nutritional deficiency, pain score, TMJ dysfunction	ORIF is favored	ORIF is favored in CF with moderate displacement between the fracture segments, with the condyle still seated in the fossa.	3b
A. Karan (2019) [25]	Prospective study	Patients with moderately displaced (neck and subcondylar) fractures	20 (10 ORIF, 10 CR)	Angulation of 10–45° and ≥2 mm ramus shortening	Specified	MO, MIO, pain scores, ramus height restoration, ROM	ORIF is favored	ORIF is favored in case of a fracture with angulation of 10 ≥ ° and or 2 ≥ mm ramus shortening.	2b
A. Kolk (2020) [19]	Prospective study	Patients with condylar head fractures	80 (54 ORIF, 26 CR)	No clear indication criteria reported	Insufficiently specified	Helkimo dysfunction index, pain scores, ROM, TMJ function	ORIF is favored	ORIF is favored except in fractures with no/mild dislocation in combination with stable occlusion.	3b
S. K. Singh (2022) [17]	Randomized comparative study	Patients with subcondylar fractures	40 (CR)	Displaced fracture, dislocation of TMJ	Insufficiently specified	Complications, MO, MIO, pain scores, ROM	N/A	CR is favored unless in displaced and dislocated fractures out of fossa.	2b
M. Kumar (2022) [68]	Retrospective study	Patients with CFs	100 (50 ORIF, 50 CR)	Drop-back test suggested by Ellis III, AAOMS and ASIF criteria, displacement > 10°	Insufficiently specified	MO, MIO, ROM, TMJ dysfunction	ORIF is favored	ORIF is favored for premature occlusal contacts due to ramus height shortening, bilateral CF, >10° of displacement, and positive drop-back test.	4
J. Lee [69] (2022)	Retrospective study	Patients with CFs	198 (103 ORIF, 95 CR)	No clear indication criteria reported	Specified	MO, MIO, ramus height restoration, tmj dysfunction	No significant difference	-	2b
J.-W. Lee (2010) [34]	Retrospective study	Patients with CFs with reduced posterior mandibular height/premature contact of molars/malocclusion	23 (ORIF)	Subcondylar fracture with proximal segment ≥ 2 cm	Clearly specified	Angulation, erosion, MO, morbidity, pain scores, resorption	N/A	ORIF is favored for patients with CFs at a low level (subcondylar fracture, proximal segment 2 cm)	4
F.-L. Merlet (2018) [70]	Retrospective study	Patients above 15 years with CFs with articular impact according to Mercier classification	83 (28 ORIF, 55 CR)	Displaced or dislocated fractures leading to loss of posterior mandibular height	Specified	Condylar remodeling, lateral excursions, MIO, ramus height restoration, symmetry	CR is favored	ORIF is preferred when the loss of height of the ramus is challenging to restore or causes occlusal disturbances.	4
P. A. Patel (2021) [71]	Retrospective study	Adult patients with CFs	27 (15 ORIF, 55 CR)	Displaced CFs according to Maclennan classification or CFs with inadequately restored occlusion with CR	Clearly specified	MO, MIO, nerve dysfunction, other complications	ORIF is favored	ORIF is favored in displaced and non-displaced fractures with abnormal occlusion with CR.	2b
R. P. Sr (2022) [20]	Prospective study	Patients aged 15–50 years, minimally displaced CFs	22 (11 ORIF, 11 CR)	Minimally displaced CFs with MO, ramus shortening, edentulous jaws, other fractures, dislocation of the condyle, or one of Zide and Kent’s indications	Insufficiently specified	Deviation on mouth opening, MO, nerve dysfunction, pain scores, restoration of condylar process, ROM	ORIF is favored	ORIF is favored for minimally displaced CFs with one or more of the earlier described presentations.	3b
N. V. V. Reddy (2013) [72]	Retrospective study	Patients with CF	175 fractures (110 ORIF, 65 CR)	Inability to restore occlusion with CR	Specified	MO, nerve dysfunction, other complications	No significant difference	ORIF is favored in case of an inability to restore occlusion by CR. Absolute contraindications are condylar head fractures, irrespective of age.	3b
R. Ren (2020) [73]	Retrospective study	Patients with condylar head fractures	56 (40 ORIF, 16 CR)	Condylar head fractures with shortening of ramus	Insufficiently specified	Disc length, Helkimo index, other complications, ramus height restoration	ORIF is favored	ORIF is favored in case of condylar head fractures that are displaced and with shortening of ramus height.	2b
V. Singh (2012) [74]	Retrospective study	Patients with bilateral subcondylar fractures	44 (24 ORIF, 20 CR)	Bilateral displaced or dislocated fractures	All fractures between 0° and 46° of angulation and up to 17 mm of ramus shortening	MIO, pain scores, ROM	ORIF is favored	ORIF is favored in bilateral displaced CFs if one or more fractures is displaced.	4
V. Singh (2018) [29]	Prospective randomized study	Patients with unilateral displaced subcondylar fractures	20 (10 ORIF, 10 CR)	Unilateral subcondylar fractures, good complement of teeth, displacement > 20°, or shortening of ramus > 10 mm	Clearly described	Deviation during mouth opening, MO, nerve dysfunction, pain scores, ramal height and displacement restoration, ROM	ORIF is favored	ORIF is favored in case of unilateral subcondylar fractures with ramus shortening > 10 mm or displacement > 20°.	2b
D. Thean (2023) [57]	Retrospective study	Patients with CFs	246 (132 ORIF, 114 CR)	Fractures with displacement > 10° or shortening of mandible > 2 mm or indications of Zide and Kent	Clearly described	MO, MIO, nerve dysfunction	Indecisive	-	4
A. Vesnaver (2020) [75]	Retrospective study	Patients with dislocated CFs, treated with surgery	7 (ORIF)	Dislocated CFs	Not specified	Condylar remodeling, deviation during mouth opening, facial asymmetry, MO, MIO, other complications, pain scores	N/A	ORIF is favored in case of dislocated CFs.	4
Y. B. Virkar (2022) [21]	Prospective study	Patients with CFs	50 (40 ORIF, 10 CR)	Cases with reduced MIO, MO, ramal height shortening, gross fracture displacement	Not specified	Condylar remodeling, MO, postoperative complications	ORIF is favored	ORIF is favored for cases with reduced MIO, MO, ramal height shortening, gross fracture displacement.	3b
P. S. Yesantharao (2012) [76]	Retrospective study	Patients with CFs	21 (6 ORIF, 8 CR, 7 soft diet)	No clear indication criteria reported	Clearly specified	MO, nerve dysfunction, other complications, TMJ dysfunction	N/A	ORIF in mixed and/or permanent dentition with symphyseal–condylar fractures.	4
X. Zhang (2019) [77]	Retrospective study	Patients with diacapitular CFs	164 (30 ORIF, 132 CR)	No clear indication criteria reported	Not specified	Facial asymmetry, MO, mandibular retrusion, radiographic abnormality, TMJ function	ORIF is favored	ORIF is favored for diacapitular condylar fractures, with dislocation out of the glenoid fossa, anteromedial disc displacement, ramus shortening ≥ 5 mm.	3b
A. Bhagol (2011) [18]	Prospective study, partially randomized	Adults with unilateral subcondylar fractures with sufficient dentition to reproduce occlusal relationships	80 (38 ORIF, 42 CR)	CR for class 1 CFs (ramal height shortening < 2 mm or displacement < 10 °C) ORIF/CR based on randomization for class 2 (2–15 mm ramus height shortening or 1–35° displacement) ORIF for class 3 (ramus shortening > 15 mm or displacement > 35°)	Clearly specified and classified	Condylar remodeling, MO, nerve dysfunction, pain, ROM	No significant difference between 3 classes. Within class 2, ORIF was favored	ORIF is favored in class 2 and 3 unilateral subcondylar fractures.	2b
T. A. Hakim (2018) [24]	RCT	Patients with unilateral subcondylar/condylar neck fractures	30 (15 ORIF, 15 CR)	Angulation 10–45° or ramus height shortening of 2–15 mm	Clearly specified	Condylar remodeling, MO, MIO, pain scores, ROM	ORIF is favored	ORIF is favored for unilateral condylar neck and subcondylar fractures in case of angulation ≥ 10° or ramus height shortening ≥ 2 mm.	1b
S. M. Kotrashetti (2013) [26]	Prospective randomized study	Adult patients with displaced CFs	22 (10 ORIF, 12 CR)	Displaced fractures according to Spiessl and Schroll classification, abnormal occlusion	Insufficiently specified	MO, deviation on mouth opening, MIO, nerve dysfunction, pain scores, ROM, TMJ function	ORIF is favored	ORIF is favored for displaced subcondylar fractures and in cases of abnormal occlusion.	2b
R. E. Rikhotso (2017) [27]	RCT	Adult patients with CFs	116 (58 ORIF, 58 CR)	No indication on forehand, results are interpreted afterwards	Wit’s classification system	MO, modified version of Helkimo’s index, MIO, pain scores, ROM, TMJ function	No significant difference	Validated mandibular condyle scoring tool to decide between ORIF and CR. Shortening of ramus height of ≥2 mm is not enough for ORIF. ORIF always in bilateral CFs.	1b
V. Singh (2010) [28]	RCT	Patients with unilateral subcondylar fractures, with sufficient dentition to reproduce occlusal relationships	40 (20 ORIF, 20 CR)	Angulation of 10–35° or ramus height shortening of >2 mm	Clearly specified	Condylar remodeling, MO, MIO, nerve dysfunction, pain scores, ROM	ORIF is favored	ORIF is favored for unilateral subcondylar fractures in case of ramus height shortening of >2 mm or angulation of >10°.	1b
Z. Zhou (2018) [78]	Retrospective study	Patients with CFs	339	CFs with angulation of 30° or more, displacement of the condyle into the fossa cranii media, or the presence of associated mid-facial fractures	Clearly specified	Condylar remodeling, MIO, ROM, TMJ function	ORIF is favored	ORIF is favored.	3b
A. K. Danda (2010) [22]	RCT	Adults with displaced unilateral subcondylar and condylar neck fractures	32 (16 ORIF, 16 CR)	Angulation between 10° and 45°	Clearly specified	Condylar remodeling, MO, MIO, pain scores, ROM	No significant difference	-	1b

CF: condylar fracture; CR: closed reduction; MO: malocclusion; MIO: maximal inter-incisal opening; N/A: not applicable; ORIF: open reduction and internal fixation; RCT: randomized controlled trial; ROM: range of motion; TMJ: temporomandibular joint.

**Table A3:** Overview of the methods and study designs of all prospective studies that aimed to validate an indication for ORIF in CFs.

Author and Year	Study Type	Inclusion Criteria for Participation in Study	ORIF Criteria	Study Population	Overview of Sample Size and Distribution in Treatment Groups	CF Fracture Type	CR Therapy Type	Follow-Up Duration and Loss to Follow-Up Reported
N. Dayalan (2021) [23]	Prospective Randomized Study	Patients within the age group of 18–60 years, diagnosed with isolated unilateral subcondylar fractures	Random Allocation	Adult	20 (ORIF 10, CR 10)	CFs with moderate displacement and the condyle still seated in the fossa	Not specified	6 months
A. Karan (2019) [25]	Prospective Randomized Study	Patients over 18, having CFs with 10–45° displacement, OR shortening of ramus height by ≥2 mm	Random Allocation	Adult	20 (ORIF 10, CR 10)	Displacement 10–45° or ≥ 2 mm ramus height shortening	Not specified	6 weeks
A. Kolk (2020) [19]	Prospective Study	Subjects without complex mandibular fractures, who were not edentulous or compliant	The decision regarding CR versus ORIF was made by the patients themselves.	Adults and adolescents	102 (ORIF 73, CR 29)	Not specified	MMF: 7-day semi-rigid MMF followed by guiding elastics; exercises started on day 7	28.5 months (mean follow-up)
R. P. Sr (2022) [20]	Prospective Study	Minimally displaced CFs, MO/ramus shortening/edentulous/other fractures/dislocation/Zide and Kent’s indications	Not reported	Adults and pediatrics	22 (ORIF 11, CR 11)	Not specified	MMF: arch bar and MMF under post-op, intermaxillary fixation with elastics changed weekly	6 months
V. Singh (2018) [29]	Prospective Randomized Study	Unilateral subcondylar fracture, ≥20° of displacement, ≥10 mm ramus shortening	Random Allocation	Adults	20 (ORIF 10, CR 10)	Displacement > 20° or ≥10 mm of ramus shortening	MMF: 7–42 days with elastics + physiotherapy	6 months
Y. B. Virkar (2022) [21]	Prospective Study	Patients that were not edentulous and without comminuted fractures	ORIF indicated in case of reduced MIO, with MO, ramus height shortening and grossly displaced fragments	Adults	50 (ORIF 40, CR 10)	Not specified	Not specified	3 months
A. Bhagol (2011) [18]	Prospective partially randomized study	Patient over 18, with unilateral subcondylar fractures and sufficient dentition to reproduce occlusal relationships	Displacement ≥ 10° or ≥2 mm ramus height shortening	Adults	80 (ORIF 38, CR 42)	Proposed their own classification	MMF: with elastics for 7–35 days. Guiding elastics used afterward for variable periods to maintain occlusion and facilitate mouth opening	6 months
T. A. Hakim (2018) [24]	RCT	Patients with displaced fractures, with angulation 10–45° and 2–15 mm of ramus shortening	Random Allocation	Adults and adolescents	30 (ORIF 15, CR 15)	Displacement and anatomical location of fracture	MMF: for 4 weeks, which was extended if needed	6 months
S. M. Kotrashetti(2013) [26]	Prospective Randomized Study	Patients over 18, with displaced CFs	Random Allocation	Adults	32 (ORIF 10, CR 12)	Displaced CFs	MMF: arch bars for initial alignment, followed by elastic MMF for 2–3 days, then switched to wires for rigid IMF for 3–4 weeks	6 months
V. Singh (2010) [28]	RCT	Patients with ramus shortening > 2 mm, angulation between 10° and 35° and sufficient dentition to reproduce the occlusal relationship	Random Allocation	Adults	40 (ORIF 20, CR 20)	Angulation 10–35°, 2 mm of ramus shortening	MMF: 7–35 days of MMF with elastics (mean, 20 days)	6 months
R. E. Rikhotso (2017) [27]	RCT	Patients with CFs, classified according to Wit’s classification	Random Allocation	Adults	116 (ORIF 48; CR 68)	After inclusion, the angulation and ramus height shortening were measured, and CFs were classified according to Wit’s classification	MMF for 1 week, followed by guided elastics for 4 weeks and physiotherapy for 3 months	12 months
A. K. Danda (2010) [22]	Prospective Randomized Clinical Study	Patients with unilateral condylar neck or subcondylar fractures with 10–45° displacement	Random Allocation	Adults	32 (ORIF 16; CR 16)	Condylar neck, subcondylar fractures, displacement of 10–45°	MMF for 2 weeks, followed by guided elastics for 2 weeks	21.9 months
S. Singh (2022) [17]	Prospective Randomized Comparative Study	Patients with CFs	Random Allocation	10 years or older	50 (ORIF 25; CR 25)	Not specified	MMF, not further specified	6 months

CF: condylar fracture; CR: closed reduction; MO: malocclusion; MIO: maximal inter-incisal opening; ORIF: open reduction and internal fixation; MMF: maxillomandibular fixation.

**Table A4:** Overview of the methods and study designs of all included systematic reviews that aimed to validate an indication for ORIF in CFs.

Author and Year	Aim and Objectives	Databases	Study Types Included	No. of Studies	Sample Size	Studies Included from Year	Clear Definition of CF for the Included Study
V. Arya (2016) [79]	To develop an algorithm for the management of intracranial condylar intrusion injuries	PubMed, Cochrane Library	Case reports, case series, reviews	62	51	Not specified	CF displaced into middle cranial fossa
A. Alyahya (2020) [80]	To review all SRs of ORIF vs. CR CFs and to propose a management algorithm	PubMed, Cochrane Library, DARE	Systematic reviews, meta-analysis	2	Not specified	Before January 2019	Not specified
R. N. Bera (2022) [56]	To compare the efficacy of CR vs. ORIF vs. endoscopic-assisted management	PubMed, Cochrane Library, ClinicalTrials.gov	RCTs	11	580 patients	1946–2020	Not specified
X. Han (2020) [81]	To evaluate the efficacy of ORIF vs. closed treatment of unilateral, moderately displaced CFs	PubMed, Embase, Cochrane Library	RCTs	6	227 patients	2008–2018	Unilateral moderately displaced condylar fracture (10–45°) or >2 mm ramus shortening
H. E. Jazayeri (2023) [82]	To evaluate quality of evidence and compare functional outcomes following ORIF vs. CR	PubMed, Embase, Cochrane Library, Scopus, Elsevier mining tool database and ClinicalTrials.gov	Meta-analyses, RCTs, non-randomized trials	14	Not specified	Not specified	CF with displacement in combination with other facial fractures
A. Kyzas (2012) [52]	To evaluate the evidence regarding the treatment that can be used for CFs	MEDLINE, Embase, Cochrane Library	RCTs, non-experimental studies	20	1186 patients	1990–2010	Not specified
J. Li (2019) [83]	To compare ORIF and CR for unilateral extracapsular CFs	PubMed, Embase, Cochrane Library	RCTs, retrospective and prospective cohorts	14	Not specified	1994–2015	Displaced/non-displaced CFs
E.A. Al-Moraissi (2015) [9]	To compare clinical outcomes between ORIF and CT for CFs	PubMed, Cochrane Library, MEDLINE, Embase, CINAH, Electronic Journal Center	RCTs, controlled clinical trials, retrospective studies	23	1318 patients	1994–2013	Not specified
B. R. Chrcanovic (2015) [10]	To evaluate differences in the incidence of post-treatment complications for CFs treated surgically or non-surgically	PubMed, Web of Science, Cochrane	RCTs, clinical studies, retrospective studies	36	1982 patients	1990–2013	Not specified
G. Minervini (2023) [7]	To evaluate the (contra)indications in the literature for the treatment of CFs via the surgical and non-surgical approach	PubMed, Web of Science, LILACS	All studies providing interventions for closed CR/ORIF treatment of CFs	4	54 patients	2000–2020	Not specified
A. Rozeboom (2017) [84]	To examine current ORIF modalities and outline the outcome measures and to align them with a recently published review on CR	PubMed, Medline, Embase	All prospective and retrospective clinical studies reporting data on unilateral CFs	70	3052 patients	1980–2016	Unilateral CF
M. Shikara (2023) [85]	To review the current literature on the treatment of subcondylar fractures (ORIF, CR, MMF and endoscopic)	PubMed, Embase, Cochrane CENTRAL, ClinicalTrials.gov, and WHO ICTRP	All studies describing management of subcondylar fractures	32	1010 patients	2001–2021	Subcondylar fractures

CF: condylar fracture; CR: closed reduction; ORIF: open reduction and internal fixation; RCT: randomized controlled trial.

**Table A5:** Overview of the outcomes of all prospective studies that aimed to validate an indication for ORIF in CFs.

Author and Year	Occlusion	Mandible Deviation (and Symmetry Parameter)	MMO	ROM (Protrusion, Laterotrusion, Mediotrusion, etc.)	Nerve Dysfunction	Ramal Height Restoration	TMJ Dysfunction	Pain Score	Function/MFIQ/Helkimo	Esthetic Improvement/Scar Formation
N. Dayalan (2021) [23]	+	+	+	+	*	ND	ND	ND	ND	*
A. Karan (2019) [25]	+	*	+	+	ND	*	ND	+	ND	ND
A. Kolk (2020) [19]	+	+	+	+	ND	ND	+	ND	+, Helkimo	ND
R. P. Sr (2022) [20]	+	+	+	+	=	ND	ND	+	ND	ND
V. Singh (2018) [29]	+	+	+	+	ND	+	ND	+	ND	ND
Y. B. Virkar (2022) [21]	+	ND	+	ND	ND	+	ND	ND	ND	ND
A. Bhagol (2011) [18]	=	+	ND	+	ND	+	ND	+	ND	ND
T. A. Hakim (2018) [24]	ND	ND	+	+	ND	+	ND	+	ND	ND
S. M. Kotrashetti (2013) [26]	+	+	+	+	*	+	ND	+	ND	ND
V. Singh (2010) [28]	=	ND	+	+	=	+	ND	+	ND	ND
R. Rikhosto (2017) [27]	+	+	=	Protrusion: = Laterotrusion to fractured side: * Laterotrusion to non-fractured side: =	*	ND	=	=	+, Dysfunction index	*
K. Danda (2010) [22]	=	=	=	=	=	+	ND	=	ND	ND
S. Singh (2022) [17]	ND	ND	ND	ND	ND	ND	ND	ND	ND	ND

Key: +: significant for ORIF, *: significant for CR, =: no statistically significant difference between ORIF and CR, ND: not described, not investigated or not reported, N/A: not applicable.

**Table A6:** Overview of the outcomes of all systematic reviews that aimed to validate an indication for ORIF in CFs.

Author and Year	Malocclusion	Mandible Deviation (and Symmetry Parameter)	MMO	ROM (Protrusion, Laterotrusion, Mediotrusion, etc.)	Nerve Dysfunction	Ramal Height Restoration	TMJ Dysfunction	Pain Score	Function/MFIQ/Helkimo/FACE-Q	Esthetic Improvement/Scar Formation
Varun Arya (2016) [79]	ND	ND	ND	ND	ND	ND	ND	ND	ND	ND
A. Alyahya (2020) [80]	+	+	+	+	*	ND	ND	+	ND	ND
R. N. Bera (2022) [56]	=	+	=	=	ND	ND	ND	+	ND	ND
X. Han (2020) [81]	=	ND	+	+	ND	ND	ND	+	ND	ND
H. E. Jazayeri (2023) [82]	+	+	ND	+	=	ND	+	+	ND	ND
A. Kyzas (2012) [52]	N/A	N/A	N/A	N/A	N/A	N/A	N/A	N/A	N/A	N/A
J. Li (2019) [83]	+	+	+	+	ND	ND	ND	=	ND	ND
Al-Moraissi (2015) [9]	+	+	+	+	ND	ND	+	=	ND	ND
B. R. Chrcanovic (2015) [10]	+	+	+	+	ND	ND	ND	=	ND	ND
G. Minervini (2023) [7]	+	+	=	+	ND	ND	=	ND	+, Helkimo	ND
A. Rozeboom (2017) [84]	N/A	N/A	N/A	N/A	N/A	N/A	N/A	N/A	N/A	N/A
M. Shikara (2023) [85]	+	+	+	+	ND	+	+	+	=	ND

Key: +: significant for ORIF, *: significant for CR, =: no statistically significant difference between ORIF and CR, ND: not described, not investigated or not reported, N/A: not applicable.

**Table A7:** Overview of the treatment protocols, authors’ proposed indications for ORIF and the level of evidence in the included prospective studies that aimed to validate an indication for ORIF in CFs.

Author and Year	Interpretation of Outcomes by Authors Favoring ORIF/CR	Proposed Indication(s) for ORIF Based on Findings	Specified an Approach, Algorithm or Management for ORIF	Limitations	Oxford Clinical Medicine Level of Evidence
N. Dayalan (2021) [23]	ORIF	Isolated low unilateral subcondylar fracture with moderate displacement between the fracture segments, but the condyle was still seated in the glenoid fossa	No	Short follow-up period, small sample size	2b
A. Karan (2019) [25]	ORIF	Displacement 10–45° or ≥2 mm ramus height shortening	No	Relatively small sample size, procedures performed by different surgeons	2b
A. Kolk (2020) [19]	ORIF	Always ORIF, unless in case of a fracture with no/mild displacement combined with stable occlusion	No	Participant’s choice of ORIF vs. CR, lack of clear indication criteria, no distinction of CF types and insufficient description of the study population in methods	1b
R. P. Sr (2022) [20]	ORIF	ORIF in case of displaced and dislocated fractures, with ramus height shortening and occlusal disharmony	No	Small sample size, solely radiographic exams on follow-up	2b
V. Singh (2018) [29]	ORIF	Displacement >20° or ≥10 mm of ramus height shortening	No	Short follow-up period, classification system not landmark-based	1b
Y. B. Virkar (2022) [21]	ORIF	ORIF indicated in case of reduced MIO, with MO, ramal height shortening and grossly displaced fragments	No	Small sample size, CR therapy unclear, no standardized indication for ORIF	2b
A. Bhagol (2011) [18]	ORIF	Displacement ≥ 10° or ≥2 mm ramus height shortening	No	Uncertain generalizability for the proposed classification	1b
T. A. Hakim (2018) [24]	ORIF	Condylar neck and subcondylar fractures with angulation ≥ 10° or ramus height shortening ≥ 2 mm	No	Not specified, no heterogeneity analysis	1b
S. M. Kotrashetti (2013) [26]	ORIF	Displaced subcondylar fractures and in case of abnormal occlusion	No	No heterogeneity analysis, small sample size	2b
V. Singh (2010) [28]	ORIF	Angulation 10–35°, ≥2 mm of ramus height shortening	No	Short follow-up period	3b
R. Rikhosto (2017) [27]	-	Defined risk factors to develop complications of CFs treated with CR; based on these, they defined a scoring system. Risk factors are bilateral fractures, ramus height shortening ≥ 5 mm, type III Wits CF fracture or higher, displacement of >15°. These do not account for condylar head fractures.	Classification of all risk factors according to severity was established, and a scoring system was developed with recommendations for either CR or ORIF.	Short follow-up period, quantification of occlusion is relative, disproportionate distribution between closed and open treatment patients	1b
K. Danda (2010) [22]	ORIF was only favored to restore anatomical relations	Displacement of 10° or more in condylar neck and subcondylar fractures	No	No heterogeneity analysis, relatively short follow-up period	2b
S. Singh (2022) [17]	ORIF is favored, based on results that are not shown	Displaced or dislocated fractures	No	No heterogeneity analysis, relatively short follow-up period	2b

CF: condylar fracture; CR: closed reduction; MO: malocclusion; MIO: maximal inter-incisal opening; ORIF: open reduction and internal fixation.

**Table A8:** Overview of the treatment protocols, authors’ proposed indications for ORIF, the level of evidence and AMSTAR-2 tool assessment in the included systematic reviews that aimed to validate an indication for ORIF in CFs.

Study	Outcomes Favoring ORIF/CR	Proposed Indication(s) for ORIF Based on Findings	Specified an Approach, Algorithm or Management for ORIF	Limitations	Oxford Clinical Medicine Level of Evidence	AMSTAR-2 Tool Assessment
Varun Arya (2016) [79]	N/A	Condylar intrusion into middle cranial fossa; co-existing CF; comminuted fracture; high risk of middle meningeal artery or brain tissue laceration; bony interference between condylar head and temporal bone; unable to reduce with closed reduction; late diagnosis (longer than 2 weeks)	Developed an algorithm for management based on indications	Low-evidence studies utilized in SR, high heterogeneity among study designs, ORIF outcomes poorly investigated.	3a	Critically low
A. Alyahya (2020) [80]	ORIF is favored	Dislocated, displaced CFs where occlusion cannot be reduced non-surgically	Developed an algorithm for the management of condylar fractures based on the displacement, dislocation of CF, occlusion state and patient-centered factors	Very few studies were included. The study does not separately describe outcomes.	1a	Moderate
R. N. Bera (2022) [56]	Both treatments were favored equally	Unilateral or bilateral, <10° displaced fractures; bilateral CFs or displacement >10°, or ramus shortening of ≥2 mm	No	Not based on specific site of CF.	1a	Moderate
X. Han (2020) [81]	ORIF is favored	Dislocated CFs; CFs displaced >45° with reduced posterior ramus height and unstable occlusion; unilateral, moderately displaced (10–45°) CFs	No	Not based on specific site of CF, sample size too small.	1a	High
H. E. Jazayeri (2023) [82]	ORIF is favored	Condylar neck/subcondylar fractures	No	High heterogeneity among study designs and outcomes, dearth of RCT data.	2a	High
A. Kyzas (2012) [52]	ORIF is favored	Not specified	No	High heterogeneity among outcomes	2a	Critically low
J. Li (2019) [83]	ORIF is favored	Not specified	No	High heterogeneity among study designs and outcomes	2a	High
Al-Moraissi (2015) [9]	ORIF is favored	Not specified	No	Heterogeneous study designs included in meta-analysis, heterogenous outcomes.	2a	Moderate
B. R. Chrcanovic (2015) [10]	ORIF is favored.	Not specified	No	Heterogeneous study designs included in the study.	2a	Moderate
G. Minervini (2023) [7]	ORIF is favored	Not specified	No	High heterogeneity among study designs and outcomes.	2a	Low
A. Rozeboom (2017) [84]	N/a	Not specified, only mentioned the used indications: malocclusion or inability to restore occlusion with CR, MMF not possible, fracture displacement and ramus height shortening. No evidence-based conclusion could be obtained.	No	Heterogeneous study designs included in the study.	2a	Critically low
M. Shikara (2023) [85]	N/a	Not specified, only mentioned indications in the studies No evidence-based conclusion could be obtained	No	Heterogeneous study designs included in the study, small sample sizes.	2a	Critically low

CF: condylar fracture; CR: closed reduction; N/A: not applicable; ORIF: open reduction and internal fixation.
